# Heat Shock Transcription Factor 2 Promotes Mitophagy of Intestinal Epithelial Cells Through PARL/PINK1/Parkin Pathway in Ulcerative Colitis

**DOI:** 10.3389/fphar.2022.893426

**Published:** 2022-07-04

**Authors:** Hao Liang, Fengrui Zhang, Wen Wang, Wei Zhao, Jiao Zhou, Yuran Feng, Jing Wu, Maojuan Li, Xinyu Bai, Zhong Zeng, Junkun Niu, Yinglei Miao

**Affiliations:** ^1^ Kunming Medical University, Kunming, China; ^2^ Department of Gastroenterology, The First Affiliated Hospital of Kunming Medical University, Kunming, China; ^3^ Yunnan Province Clinical Research Center for Digestive Diseases, Kunming, China; ^4^ Department of Ultrasound, First Affiliated Hospital of Kunming Medical University, Kunming, China; ^5^ Organ Transplantation Center, The First Affiliated Hospital of Kunming Medical University, Kunming, China

**Keywords:** heat shock transcription factor 2, mitophagy, intestinal epithelial cells, ulcerative colitis, pathogenesis

## Abstract

The overactivation of NLRP3 inflammasome in intestinal epithelial cells (IECs) is among the important reasons for severe inflammation in ulcerative colitis (UC). We found that heat shock transcription factor 2 (HSF2), which is highly expressed in UC, could inhibit the activation of NLRP3 inflammasome and reduce IL-1β in IECs, but the mechanisms were still not clear. It has been reported that HSP72 regulated by HSF2 can enhance the mitophagy mediated by Parkin. The number of damaged mitochondria and the mitochondrial derived ROS (mtROS) can be reduced by mitophagy, which means the activity of NLRP3 inflammasome is inhibited. Therefore, we speculate that HSF2 might regulate the activation of NLRP3 inflammasome of IECs in UC through the mitophagy mediated by Parkin. This study proves that the number of damaged mitochondria in IECs, the level of mitophagy, and the level of ROS in intestinal mucosa are positively correlated with the severity of UC. In mice and cells, mitophagy was promoted by HSF2 through the PARL/PINK1/Parkin pathway. This study reveals the potential mechanisms of HSF2 decreasing mtROS of IECs in UC.

## Introduction

Ulcerative colitis (UC) is a chronic, recurrent inflammatory disease. In recent years, the incidence of UC is growing rapidly around the world, especially in Asia, including China (Kaplan and Ng, 2017; Ng et al., 2017). Although previous studies have shown that the occurrence of UC is related to genetic susceptibility, immune overreaction, intestinal flora disorder, and an urban lifestyle, its exact etiology and pathogenesis are still unclear (Ordás et al., 2012; Zuo et al., 2018). Due to the unclear pathogenesis of UC and the lack of effective therapeutic drugs, most UC patients still have repeated disease activity, although various biological agents have shown some improved efficacy. Therefore, there is an urgent need to explore the pathogenesis of UC and find new therapeutic targets.

In recent years, studies have shown that a moderate inflammatory response is an important part of the intestinal mucosa to resist the invasion of pathogenic microorganisms and maintain the homeostasis of intestinal epithelial cells, but excessive and continuous inflammation is a critical factor in the occurrence and development of UC (Sánchez de Medina et al., 2014; Yao et al., 2019). Innate immune disorder of the intestinal mucosa has become one of the important characteristic markers of UC (Geremia et al., 2014). It has been reported that the NLRP3 inflammasome, an indispensable component of innate immunity, plays a vital role in innate immune response and disease occurrence (Próchnicki and Latz, 2017). Proper activation of NLRP3 inflammasome is fundamental for the body to fight against microbial infection and regulate the mucosal immune response (Strowig et al., 2012); however, excessive activation can cause a severe inflammatory response, damage the intestinal epithelial barrier, and participate in the process of UC by cleaving caspase1 and promoting the massive secretion of proinflammatory factors such as IL-1β (Ranson et al., 2018; Zhen and Zhang, 2019). In short, proper activation of the NLRP3 inflammasome is an important factor in maintaining intestinal mucosa and body homeostasis. Therefore, further exploration of the regulatory mechanism of the NLRP3 inflammasome in UC is significant for understanding the pathogenesis of UC.

Our team carried out a series of studies on intestinal mucositis in UC and found that HSF2, which can maintain cell homeostasis, is specifically expressed at a high level in UC and can inhibit LPS-induced IL-1β in Caco-2 cells, but the specific mechanism is unclear (Miao et al., 2014). We further found that HSF2 reduced secretion of IL-1β by inhibiting NLRP3 activation, but the specific mechanism of HSF2 inhibiting the activation of NLRP3 inflammasome is still unclear (Zhang et al., 2021).

The NLRP3 inflammasome can be activated by a variety of endogenous or exogenous stimuli in the body, including mitochondrial DNA, mtROS, and potassium ion efflux (Kelley et al., 2019; Swanson et al., 2019; Yang et al., 2019). As an important activator, the ROS level determines the activation of the NLRP3 inflammasome and the inflammation level of intestinal mucosa. Studies have reported that excessive accumulation of ROS in the intestinal mucosa is one of the characteristics of UC (Wang et al., 2016). In addition, it has been reported that both patients with UC and mice with DSS-induced colitis showed an increase of damaged mitochondria in IECs (Spoorthi et al., 2020; Chandraiah et al., 2021), and damaged mitochondria are an important source of ROS (Kudryavtseva et al., 2016). Therefore, timely removal of damaged mitochondria is essential to reduce intracellular ROS, inhibit inflammation, and maintain intestinal mucosal homeostasis.

Mitophagy is a crucial way to remove intracellularly damaged mitochondria, but its function and mechanism in UC are not well studied. As reported (Guo et al., 2014), mitophagy can regulate the activation of NLRP3 inflammasome in the intestinal mucosa of mice with DSS-induced colitis and play a protective role, which is similar to the role of HSF2 in UC. The PARL/PINK1/Parkin signaling pathway plays an important role in the regulation of mitophagy (Ashrafi and Schwarz, 2013). Studies have suggested that HSF2 regulation of HSP72 can affect the expression of Parkin protein and promote mitophagy (Kwong et al., 2006; Ostling et al., 2007; Ahn et al., 2008; Drew et al., 2014). Therefore, we speculate that HSF2 inhibits the activation of NLRP3 inflammasome of IECs in UC by regulating the level of mitophagy. In order to confirm this conjecture, we carried out this study.

In this study, we found that HSF2 in UC can enhance mitophagy, reduce intracellular ROS, inhibit the activation of NLRP3 inflammasome and relieve mucosal inflammation through the PARL/PINK1/Parkin signaling pathway, which provides a new direction for exploring the pathogenesis of UC and developing therapeutic targets.

## Materials and Methods

### Patient Selection and Sample Processing

A total of 90 UC patients were selected for the study according to the opinions of the third European Crohn’s and Colitis Organization (ECCO) consensus on the diagnosis and management of UC. The patients were treated at the First Affiliated Hospital of Kunming Medical University from January 2018 to December 2019. Disease severity was classified by the Mayo Score as mild, moderate, or severe. The all lesions were of left semicolon type and only mesalazine was used for treatment within 3 months before hospitalization (all patients who needed other drugs, such as glucocorticoids and immunosuppressants, to control the disease underwent colonoscopy and biopsy as soon as possible before medication, so as to reduce the interference of those drugs on the results of this study). At the same time, 60 healthy volunteers for colon cancer screening were chosen as controls. The required age range was between 18 and 60 years Tables 1, 2 shows the information and characteristics of enrolled participants. All colonoscopy examinations were performed by specialists in the Department of Gastroenterology, and each patient or healthy control was taken endoscopic biopsy in the sigmoid colon with 6 pieces and each 0.4–0.5 cm in diameter. All enrollees signed the informed consent form.

**TABLE 1:** Details of UC patients and healthy controls.

Patient details	Number of patients (%)	Healthy controls details	Number of healthy controls (%)
Gender			
Male	48 (53.3%)	Male	15 (50%)
Female	42 (46.7%)	Female	15 (50%)
Mean age (years)	40.5 (19–58)	Mean age (year)	42.2 (18–57)
Diagnosis		Diagnosis	
UC (Mayo score)		Healthy	
Mild	33 (36.7%)	-	-
Moderate	30 (33.3%)	-	-
Severe	27 (30%)	-	-
Actual disease extent			
E1	0 (0%)	-	-
E2	90 (100%)	-	-
E3	0 (0%)	-	-
Mayo Score			
0	0 (0)	-	-
1	33 (36.7%)	-	-
2	29 (32.2%)	-	-
3	28 (31.1%)	-	-
Medication			
5-ASA (mesalazine)	90 (100%)	-	-

**TABLE 2:** Parameters related to disease activity of UC patients.

Group	CRP [M(Min ∼ Max)]	ESR [M(Min ∼ Max)]	CAI [M(Min ∼ Max)]
Mild	4.54 (2∼37)	14.65 (0.5∼25.46)	2.3 (0∼6)
Moderate	7.60 (6∼38)	18.47 (0.45∼26.69)	4.7 (2∼11)^#^
Severe	61.27 (3∼73)*	38.91 (3∼162.50)*	8.2 (4∼12)*

*Compared with moderate UC, *p* < 0.0001.

^#^Compared with mild UC, *p* < 0.0001.

### Mice

The *hsf2* ^*−/−*^ mice were created by CRISPR/Cas9 gene Technology and purchased from the Cyagen Biosciences Inc. However, after repeated verification, it was found that *hsf2* protein of *hsf2* ^*−/−*^ mice was not completely knocked out, but the expression level of *hsf2* protein was significantly decreased compared with wild-type mice. Therefore, we define the *hsf2* ^*−/−*^ mice as knock down group (KD group). A total of 40 male mice (7–8 weeks) were divided into wild-type (WT, 20) and knock down (KD, 20) groups. Then each group was randomly divided into 2 subgroups, with 10 mice in each (WT + H_2_O, WT + DSS, KD + H_2_O, KD + DSS). The WT + H_2_O and KD + H_2_O groups were given distilled water for 7 days, and the WT + DSS and KD + DSS groups were given 3% dextran sulfate sodium (DSS) (MP Biomedicals, United States) for 7 days. The disease activity index (DAI) of all mice was measured every day. On the 8th day, all mice were killed, and colon specimens were taken to measure their length. Each mouse colon was cut into 5 segments: one was immediately put into 2% glutaraldehyde for electron microscope observation, 2 were stained with hematoxylin eosin (HE) and detected by immunohistochemistry, and 2 were stored at −80°C for RT-PCR and Western blotting.

### Cell Culture

Human colon adenocarcinoma cells (Caco-2) were used in this study. The Caco-2 cells were obtained from the cell culture library of the Kunming Institute of Zoology, Chinese Academy of Sciences. After the cells were resuscitated in a water bath at 37°C constant temperature, they were cultured in DMEM:F12 (1:1) medium containing 10% fetal bovine serum and then incubated under 5% CO_2_ at 37°C.

### Regulating HSF2 Expression by Transfecting Lentiviral

HSF2 lentiviruses of overexpression and knockdown were purchased from Shanghai GeneChem Co., Ltd. Viruses were transfected into Caco-2 cells according to the lentivirus product instructions. The MOI of Caco-2 cells was set at 20. In addition, a certain amount of HittransG P was added to the transfection group to make a final concentration of 1ug/ml, so as to improve transfection efficiency. According to the experimental method of Wang et al. (Wang et al., 2020), an appropriate amount of LPS(200 ng/ml for 6 h) and ATP (5 mmol/ml for 0.5 h) was added to the cell culture medium to stimulate inflammation.

### Transmission Electron Microscope

TEM (JEM-1400 Flash, Nippon Electronics Corporation) was provided by Kunming Medical University. The collected colonic mucosal tissues were immediately placed in 2.5% glutaraldehyde and stored at 4°C in a refrigerator. PBS was first used for repeated washing 3 times, then osmium acid (1%) was used for fixation for 2 h, and then washing again 3 times for 10 min each time. Dehydration was then carried out by ethanol and acetone (paying attention to the temperature). Finally, after handling the embedding solution, staining by uranyl acetate and lead citrate, and drying, the specimens were observed under TEM.

### Immunohistochemistry

The intestinal mucosal tissue specimens were embedded with paraffin, fixed with 4% paraformaldehyde, and sliced continuously with a slicer. They were then defatted and hydrated in a solution of xylene, then treated with a citrate buffer (0.01 M) under 800 W microwave, and incubated for 10 min in 3% hydrogen peroxide at room temperature. After that, 50 µL of primary antibody (PARL or PINK1 or Parkin) was added to the slices, followed by incubation at 4°C overnight. The antibodies used in this study were anti-HSF2(Santa Cruz, 1:200), anti-PARL (1:400, Proteintech), anti-PINK1 (1:300, Proteintech), anti-Parkin (1:250, Proteintech). Another 50 µl DAB was added for color development, and the dyeing time and degree were controlled under microscope observation. Double steam water was used to wash twice, 1 minute each time, and hematoxylin was used for restaining (1 min), followed by rinsing with 1% ammonia after removal. The slides were successively immersed in 95 and 100% ethanol, and were dehydrated twice in total. After blow drying, they were sealed with neutral resin and the results were observed under the microscope.

### Immunofluorescence

The ROS level in tissues were observed by laser confocal microscopy. First, appropriate OCT encapsulating agent was poured into the premarked specimen box. Then, the mucosal colon tissues were quickly placed in the specimen box and stored at −80°C. The second part was staining. After the frozen section was taken out, ROS dye was added, and the sample was incubated in darkness for 30 min at 37°C. Second, nuclear staining was carried out. Finally, the tablets were sealed with anti-fluorescence sealing tablets. The slices were observed under a laser confocal microscope and images were collected (blue light: DAPI ultraviolet excitation wavelength 330–380 nm, emission wavelength 420 nm; red light: CY3 excitation wavelength 510–560 nm, emission wave length 590 nm).

### Flow Cytometry

The Mitochondrial ROS detection Assay Kit (Item No.701600) was purchased from the Cayman Chemical Company (Ann Arbor, Michigan). Mitochondrial ROS level in each group were detected by flow cytometry (Beckman coulter cell, United States). Firstly, an appropriate amount of pre-warmed Cell-Based Assay Buffer was added to the Caco-2 cells with medium removal, and then 100 µl of preheated Mitochondrial ROS Detection Reagent Staining Solution was added to each well, which was incubated at 37°C and shielded from light for 20 min. After that, the staining solution was removed and an appropriate amount of pre-warmed HBSS was added to each well and washed three times, and then the cells were incubated at 37°C for 1 h. Finally, the excitation wavelength was set at 480–515 nm, and the emission wavelength was set at 560–600 nm. The mitochondrial ROS level of cells in each group were detected by flow cytometry.

### Reverse Transcription Polymerase Chain Reaction

This part of the experiment was conducted according to the Platinum® SYBR® Green qPCR kit (TaKaRa, Japan) instructions. Each part of the experiment was repeated at least 3 times. The primer sequence in this study was designed and synthesized by TaKaRa Bio Inc. as follows:

HSF2 (Forward): 5′GAA​ACC​CAC​ACT​AAC​GAG​TTC​ATC​A-3′

HSF2 (Reverse): 5′TGC​CTC​ACA​AAG​CTT​GCC​ATA-3′

PARL (Forward): 5′-CCT​ATA​AGA​ACA​CTC​GTG​AAG​CC-3′.

PARL (Reverse): 5′-CCA​GTC​AGC​TTT​TAT​GCC​ATC-3′.

PINK1 (Forward): 5′-GGT​GTC​AGG​CTG​GGG​CAA-3′.

PINK1 (Reverse): 5′-TGG​CTT​CAT​ACA​CAG​CGG​C-3′.

Parkin (Forward): 5′-TCT​TCG​GCA​TCT​TGT​CTG-3′.

Parkin (Reverse): 5′- CTG​GGA​GTC​GTA​GTT​CTA​ACG -3′.

GAPDH (Forward): 5′-CAA​GTT​CAA​CGG​CAC​AGT​CA-3′.

GAPDH (Reverse): 5′-CAC​CCC​ATT​TGA​TGT​TAG​CG-3′.

### Western Blotting

Mouse tissue samples or cells were homogenized in lysis buffer containing 1% protease inhibitors. Protein assay kit was used to determine the protein concentration. The antibodies used in this study were anti-HSF2(Santa Cruz, 1:1000), anti-PARL (1:1000, Santa Cruz), anti-PINK1 (1:1000, Santa Cruz), anti-Parkin (1:1000, Santa Cruz), anti-β-actin (1:5000, Abcam), and anti-GAPDH (1:5000, Abcam). The results were analyzed by ImageJ.

### Statistical Analysis

SPSS 25.0 software was used for statistical analysis of all data. Measurement data were expressed as mean ± standard deviation. One-way ANOVA was used for measurement of data between groups, and LSD was used for pairwise comparison between groups. In the figures, *p < 0.05, **p < 0.01, ***p < 0.001 and ****p < 0.0001.

## Results

### The Damaged Mitochondria, Mitophagy and Mitochondrial Derived ROS Increased in Intestinal Epithelial Cells of Ulcerative Colitis and That Could be Affected by Heat Shock Transcription Factor 2 *in vitro* and *in vivo* Experiments

**FIGURE 1:**
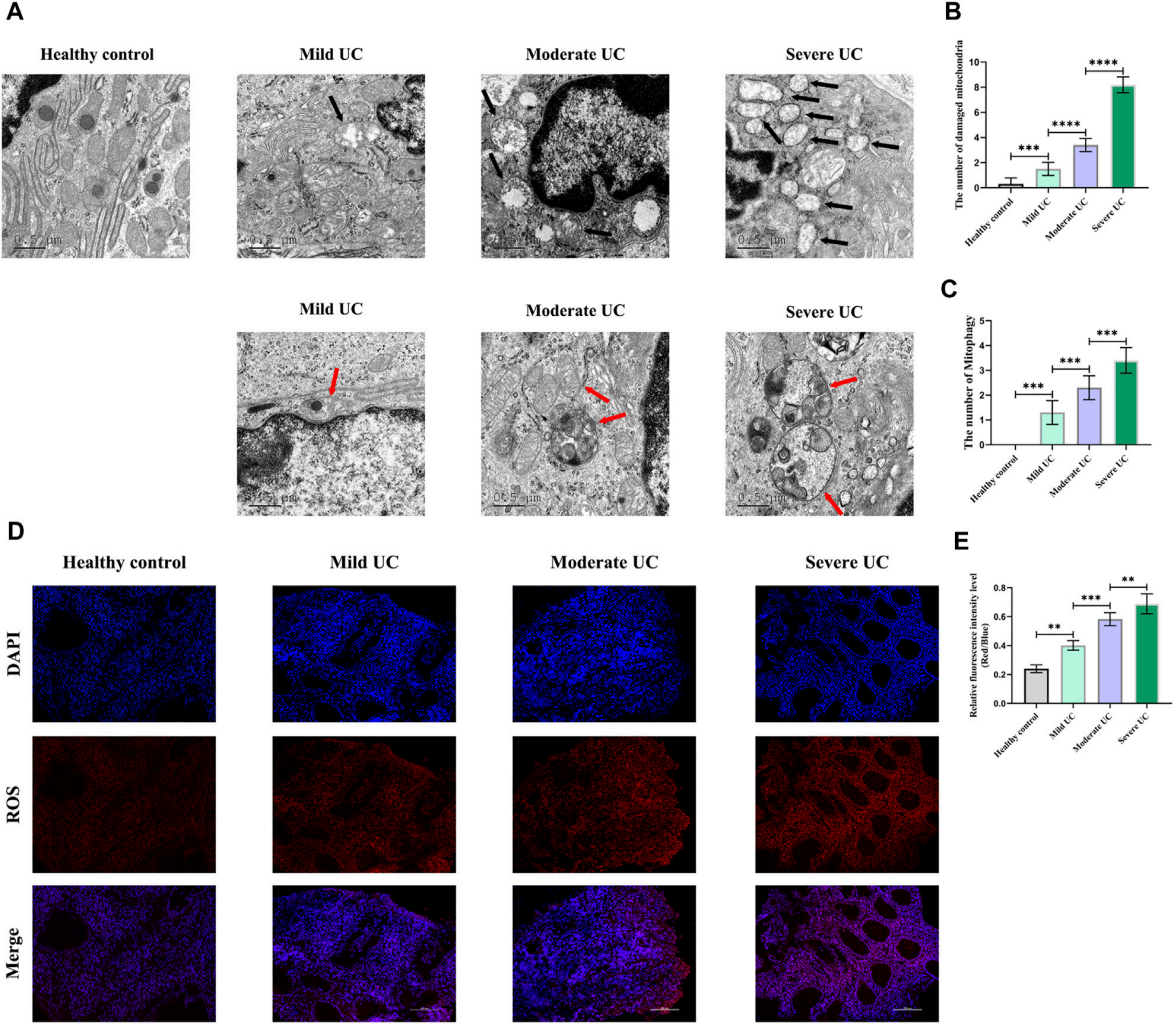
Compared with healthy controls, obvious morphological abnormalities present in mitochondria of IECs of UC patients, and mitophagy level of IECs rose with increased disease activity, showing a positive correlation. In addition, ROS level was higher in intestinal mucosal tissues of UC patients than healthy controls. With increased of disease severity, ROS level in intestinal mucosal tissues increased continuously. **(A–C)** The number of damaged mitochondria and the level of mitophagy in healthy controls and UC patients were observed by TEM. (Black arrows show damaged mitochondria: the volume of mitochondria became larger and rounded, the matrix became shallow, the cristae became shorter and less or disappeared; red arrows show mitophagy: double membrane autophagosomes containing unique mitochondrial structures including the cristae (early stage), or single membrane autolysosomes with residue mitophagy (late stage)). **(D,E)** Immunofluorescence was used to detect ROS level in intestinal mucosal tissues of healthy controls and UC patients. These data expressed as means ± standard deviation, and statistical results were obtained by one-way ANOVA. Statistical significance at **p < 0.01, ***p < 0.001 and ****p < 0.0001.

Firstly, we detected the damaged mitochondria in the IECs of normal controls and UC patients with different disease severity by TEM, and found that the number of damaged mitochondria in UC patients was significantly increased compared with the normal controls, and the number of damaged mitochondria was positively correlated with disease severity (Figure 1A,B). Secondly, immunofluorescence was used to detect the level of ROS in the intestinal mucosa of normal controls and UC patients with different disease severity. It was found that the level of ROS was significantly increased in UC patients, and was positively correlated with the disease severity (Figure 1D,E). Thirdly, we used TEM to detect mitophagy of IECs in UC patients with different disease severity and found that the level of mitophagy in UC patients of each group was significantly higher than that of normal controls, and was positively correlated with the severity of UC (Figure 1A,C). These results once again demonstrated that there was obvious mitochondrial damage in the IECs of UC patients, and a large amount of ROS accumulated in the intestinal mucosa of UC patients.

Mitophagy, as an important way to remove damaged mitochondria and mtROS, can inhibit the activation of NLRP3 inflammasome by reducing the level of mtROS, which is an important activator of NLRP3 inflammasome. The increased level of mitophagy in UC patients was also confirmed in this study. Combined with our previous studies, HSF2 expressed highly in intestinal mucosa of UC patients (Supplementary Figures S1), and can reduce IL-1β by inhibiting the activation of NLRP3 inflammasome. Therefore, we speculate that the protective effect of HSF2 on inhibiting NLRP3 inflammasome activation and intestinal mucositis may be achieved by promoting intracellular mitophagy and reducing mtROS. In order to test this hypothesis, we conducted further *in vitro* and *in vivo* experiments.

**FIGURE 2:**
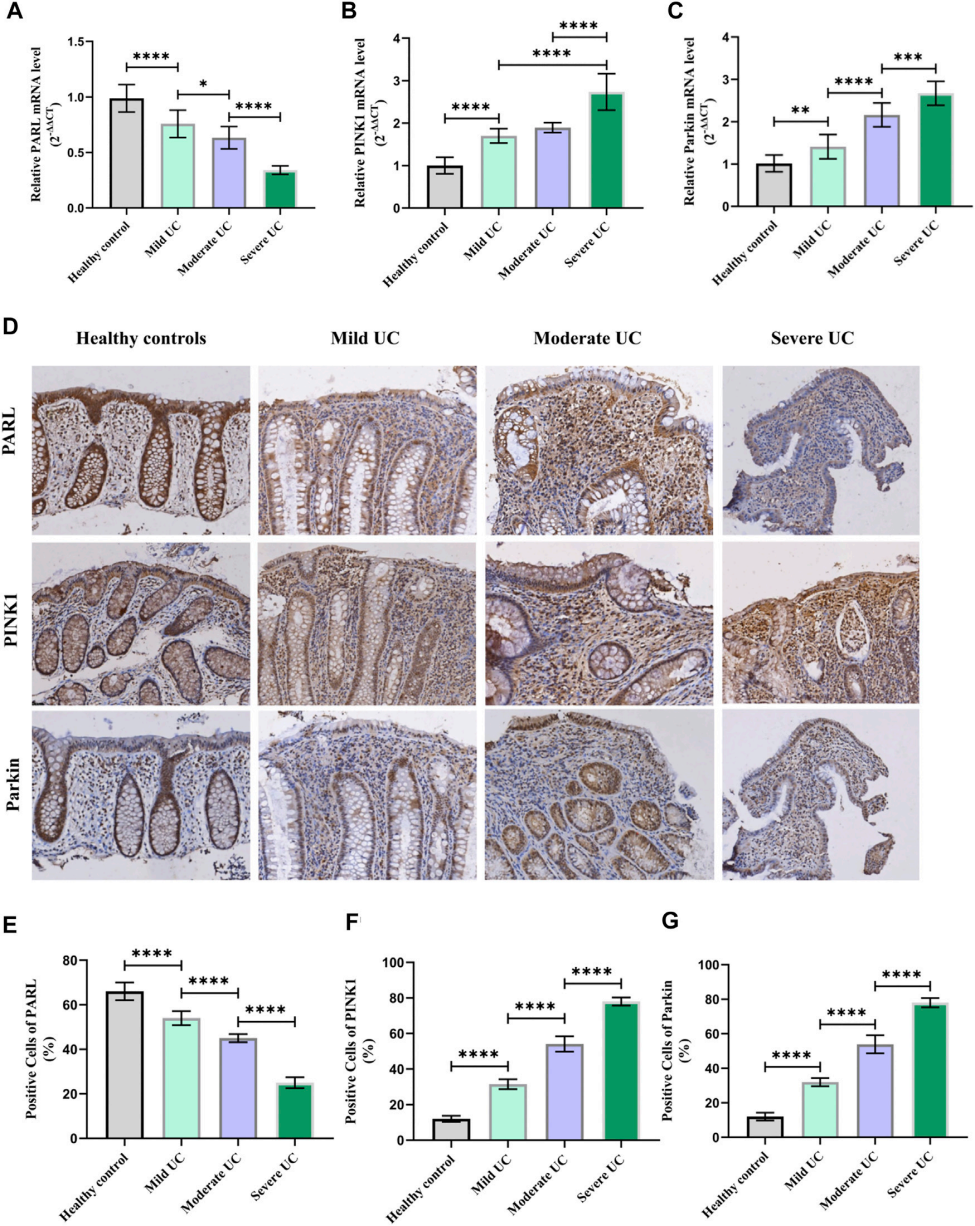
Higher severity of UC means lower PARL; PARL level in intestinal mucosa of UC patients decreased, and PINK1 and Parkin level rose. **(A–C)** Expression level of PARL, PINK1, and Parkin genes in intestinal mucosa of each group were measured by RT-PCR. **(D–G)** Immunohistochemistry was used to detect level of PARL, PINK1, and Parkin proteins in intestinal mucosa of mice. These data were expressed as means ± standard deviation, and statistical results were obtained by one-way ANOVA. Statistical significance at *p < 0.05, **p < 0.001, ***p < 0.001 and ****p < 0.0001.

DSS was used to induce colitis in *hsf2* ^*−/−*^mice and wild-type mice (Supplementary Figures S2). TEM showed that the number of damaged mitochondria in IECs of *hsf2* ^*−/−*^ colitis mice was significantly higher than that of wild-type mice, while the level of mitophagy was lower than that of wild-type mice (Figure 2A–C). In addition, immunofluorescence was used to detect ROS level in intestinal mucosa of mice in each group. Compared with wild-type mice, the level of ROS in intestinal mucosa of *hsf2* ^*−/−*^ colitis mice was significantly increased (Figure 2D,E). These results reveal an important relation between *hsf2* and mitophagy and mtROS. To further confirm this relation, we conducted *in vitro* experiments.

**FIGURE 3:**
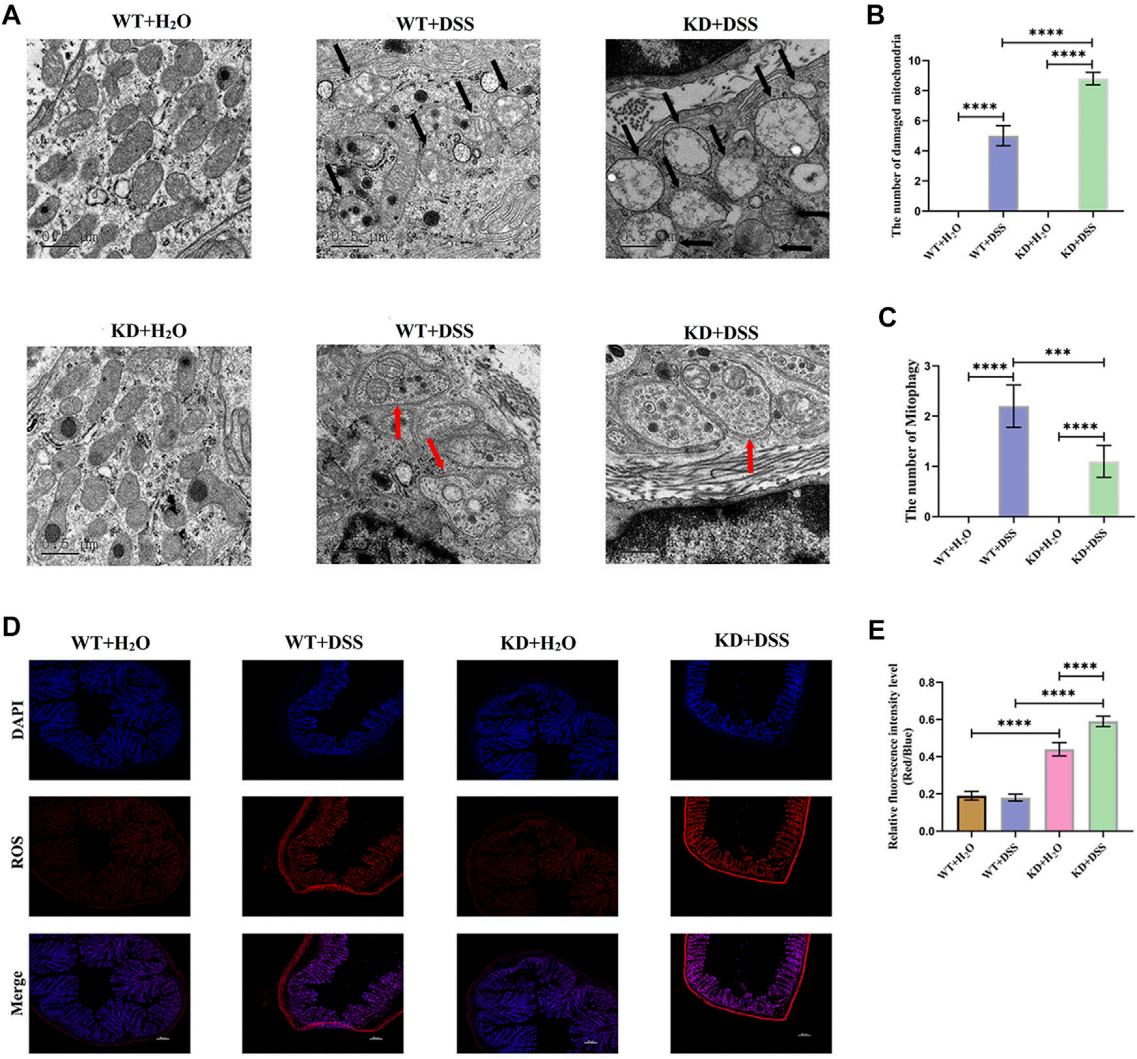
Compared with WT+DSS group, mitophagy of intestinal mucosa of KD+DSS group were less and damaged mitochondria were more obvious, and ROS level of KD+DSS group was higher. No obvious mitophagy and damaged mitochondria occurred in WT+H2O and KD+H2O groups, which were used as controls. **(A–C)** TEM was used to observe level of mitophagy and damaged mitochondria in intestinal mucosa of mice (Black arrows show damaged mitochondria: the volume of mitochondria became larger and rounded, the matrix became shallow, the cristae became shorter and less or disappeared; red arrows show mitophagy: double membrane autophagosomes containing unique mitochondrial structures including the cristae (early stage), or single membrane autolysosomes with residue mitophagy (late stage)). **(D,E)** Immunofluorescence was used to detect ROS level in intestinal mucosa of mice in each group. These data were expressed as means ± standard deviation, and statistical results were obtained by one-way ANOVA. Statistical significance at ***p < 0.001 and ****p < 0.0001.

First, we successfully regulated the expression of HSF2 in Caco-2 cells by lentivirus transfection (Supplementary Figures S3), and stimulated Caco-2 cells with LPS and ATP to generate inflammation. Next, we observed Caco-2 cells with TEM, and found that increased damaged mitochondria and mitophagy showed in the LPS and ATP stimulated Caco-2 cells; Overexpression of HSF2 could increase the level of LPS and ATP induced mitophagy and decrease the number of damaged mitochondria in Caco-2 cells. On the contrary, downregulating HSF2 could decrease the level of mitophagy and increase the number of damaged mitochondria in Caco-2 cells (Figure 3A–C). Moreover, flow cytometry showed that mtROS level in Caco-2 cells increased significantly when LPS and ATP was used to stimulate the cells, and decreased after overexpression of HSF2. On the contrary, knockdown of HSF2 could increase the level of ROS (Figure 3D,E). These results are consistent with our expectation and similar to the results of UC patients and DSS-induced colitis mice: the level of *hsf2* is positively correlated with the level of mitophagy in IECs, which suggests that mitophagy and mtROS could be affected by HSF2. However, how HSF2 affects mitophagy and mtROS needs further validation.

### The PARL/PINK1/Parkin Pathway Regulated by Heat Shock Transcription Factor 2 Might Be Critical for the Level of Damaged Mitochondria, Mitophagy and Mitochondrial Derived ROS in Intestinal Epithelial Cells of Ulcerative Colitis

**FIGURE 4:**
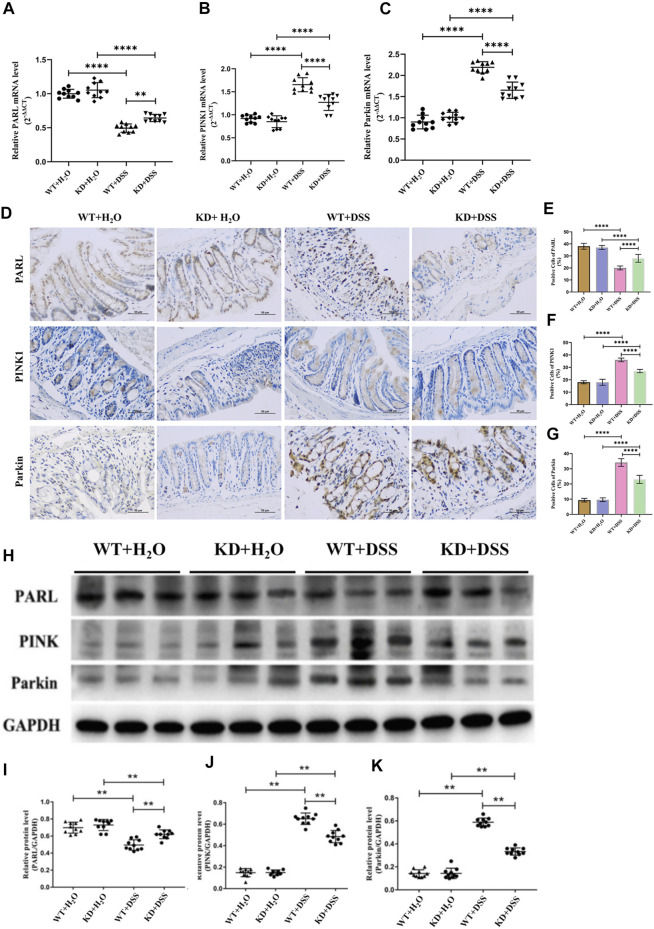
With *hsf2* gene knocked down, level of mitophagy of intestinal mucosa decreased. **(A–C)** RT-PCR was used to detect the expression of PARL, PINK1, and Parkin genes in intestinal mucosa of mice in each group. Compared with WT+DSS group, higher level of PARL gene presented in KD+DSS group, but level of PINK1 and Parkin genes were lower. **(D–G)** Immunohistochemistry was used to detect level of PARL, PINK1, and Parkin proteins in intestinal mucosa of mice in each group. Compared with WT+DSS group, level of PARL protein in KD+DSS group was higher, but level of PINK1 and Parkin protein were decreased. **(H–K)** Western blotting was used to detect level of PARL, PINK1, and Parkin proteins of intestinal mucosa of mice in each group. The results were consistent with PCR and IHC. These data of were expressed as means ± standard deviation, and statistical results were obtained by one-way ANOVA. Statistical significance at **p < 0.01 and #x2a;***p < 0.0001.

Since the PARL/PINK1/Parkin signaling pathway is the most studied mitophagy pathway at present. In order to further clarify whether the level of mitophagy in UC patients is related to this pathway, RT-PCR and IHC were used to detect the mRNA and protein expression of PARL, PINK1 and Parkin in the intestinal mucosa of UC patients and healthy controls. Compared with the healthy controls, the level of PINK1 and Parkin in the intestinal mucosa of UC patients were increased, while the level of PARL was decreased (Figure 4). In Addition, the above changes in the intestinal mucosa and IECs of UC are closely related to the severity of disease.

**FIGURE 5:**
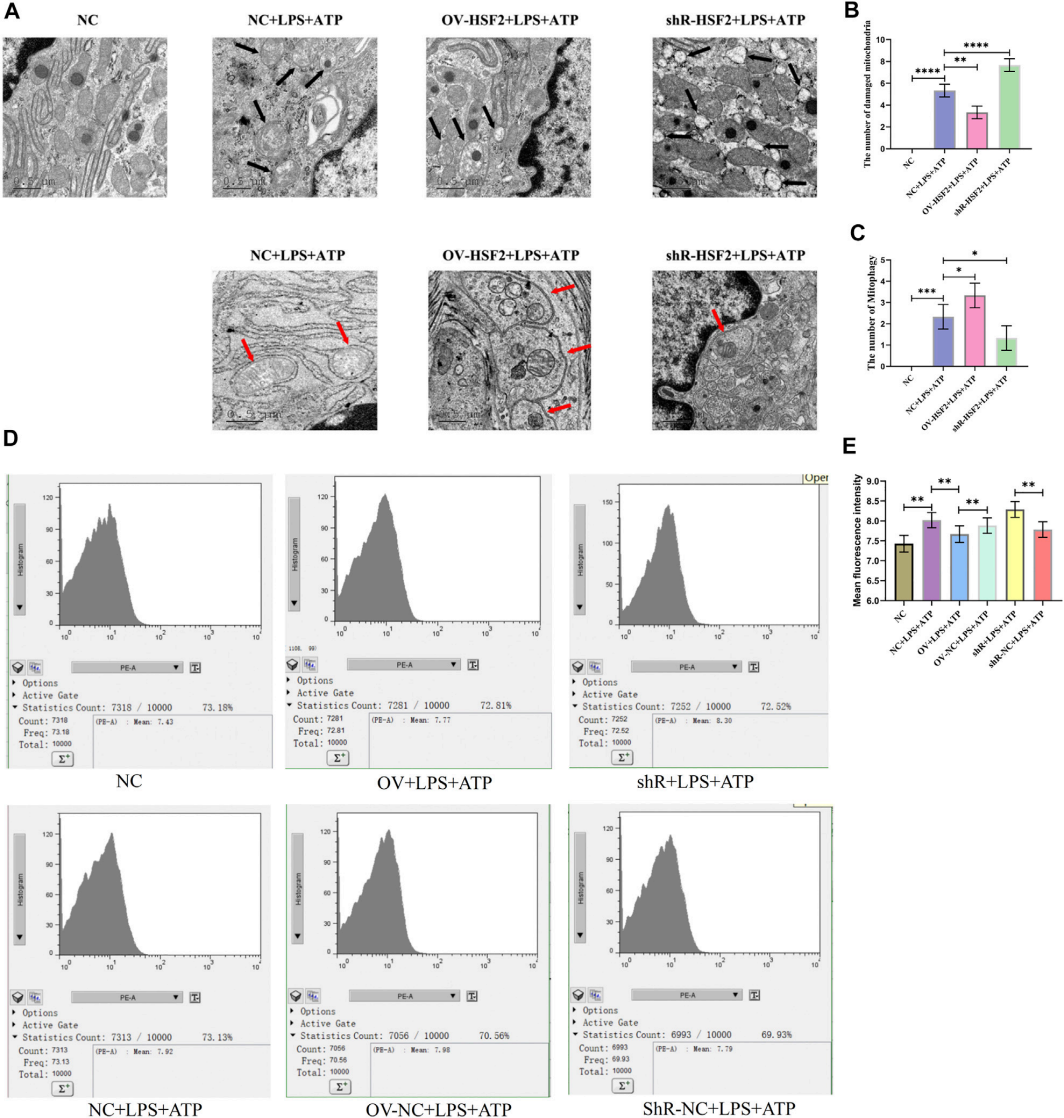
After stimulation with LPS and ATP, the number of damaged mitochondria and mitophagy in Caco-2 cells was significantly increased and intracellular mtROS level was significantly increased. Overexpression of HSF2 increased mitophagy level in Caco-2 cells and reduce number of intracellularly damaged mitochondria. Conversely, downregulation of HSF2 reduced mitophagy level in Caco-2 cells and increased number of intracellularly damaged mitochondria. In addition, overexpression of HSF2 reduced intracellular mtROS level. On the contrary, after downregulation of HSF2, intracellular mtROS level increased. **(A–C)** Mitochondrial morphology and mitophagy of Caco-2 cells in each group were detected by TEM. (Black arrows show damaged mitochondria: the volume of mitochondria became larger and rounded, the matrix became shallow, the cristae became shorter and less or disappeared; red arrows show mitophagy: double membrane autophagosomes containing unique mitochondrial structures including the cristae(early stage), or single membrane autolysosomes with residue mitophagy (late stage)). **(D,E)** Intracellular mtROS level of Caco-2 cells treated differently in each group were detected by the Mitochondrial ROS detection Assay Kit and flow cytometry. These data are expressed as means ± standard deviations, and statistical results were obtained by one-way ANOVA. Statistical significance at *p < 0.05, **p < 0.01, ***p < 0.001 and ****p < 0.0001.

*In vivo* experiment, RT-PCR, IHC and western blotting showed that compared with wild-type mice, the mRNA and protein expression level of PARL, PINK1 and Parkin in intestinal mucosa of *hsf2* ^*−/−*^ DSS colitis mice were significantly changed: the level of PARL was increased, while the level of PINK1 and Parkin were decreased (Figure 5). These results suggest that downregulating *hsf2* may reduce the level of mitophagy through the PARL/PINK1/Parkin pathway in IECs of DSS-induced colitis mice.

**FIGURE 6:**
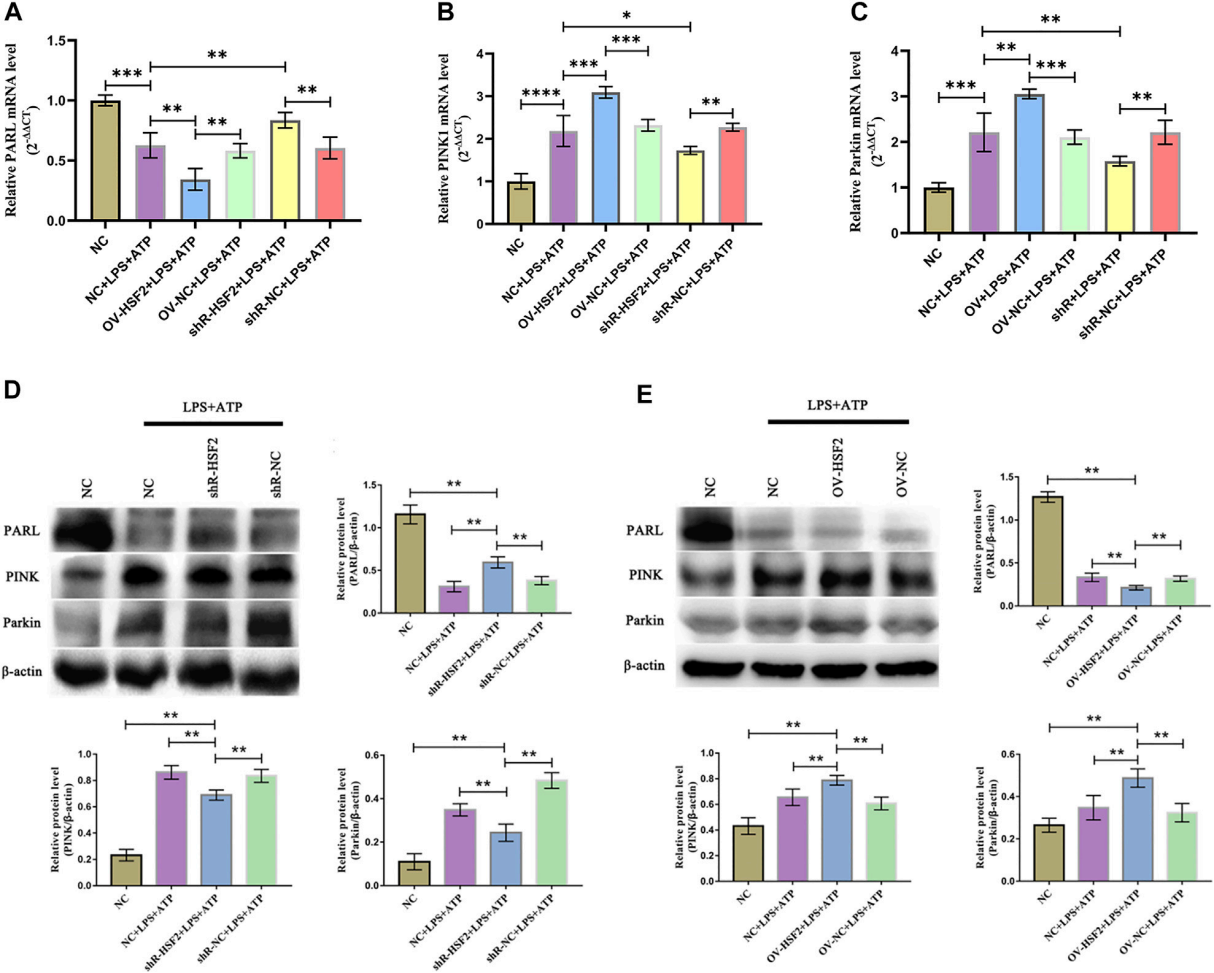
HSF2 may regulate mitophagy in Caco-2 cells through PARL/PINK1/Parkin pathway. When HSF2 overexpressed, level of PARL in Caco-2 cells decreased, and level of PINK1 and Parkin increased; Level of PARL was higher after HSF2 knockdown, and level of PINK1 and Parkin were lower. **(A–C)** RT-PCR was used to detect level of PARL, PINK1, and Parkin genes in Caco-2 cells of each group. **(D,E)** Western blotting was used to detect level of PARL, PINK1, and Parkin proteins in Caco-2 cells of each group. Results were consistent with RT-PCR. These expressed as means ± standard deviation, and statistical results were obtained by one-way ANOVA. Statistical significance at *p < 0.05, **p < 0.01, ***p < 0.001 and ****p < 0.0001.

Similar results were found *in vitro* experiments. After stimulation of LPS and ATP, the gene and protein level of PARL, PINK1 and Parkin in Caco-2 cells were significantly changed: the level of PARL was decreased, However, the level of PINK1 and Parkin increased (mitophagy was increased). Overexpression of HSF2 could further enhance the effect of LPS on the gene and protein level of PARL, PINK1 and Parkin: the level of PARL was further decreased, and the level of PINK1 and Parkin was further increased (mitophagy was decreased); On the contrary, downregulation of HSF2 attenuated the effect of LPS on the gene and protein level of PARL/PINK1/Parkin signaling pathway (Figure 6). In addition, as an important downstream molecule of NLRP3 inflammasome, we detected the level of IL-1β and IL-18 in each group by ELISA. The level of IL-1β and IL-18 increased significantly after LPS and ATP was used to stimulate the cells. Moreover, after overexpression of HSF2, the intracellular IL-1β and IL-18 decreased; On the contrary, after downregulation of HSF2, the level of IL-1β and IL-18 increased (Supplementary Figures S4).

##  Discussion

Intestinal mucosal homeostasis is the basis of maintaining normal intestinal function. Moderate inflammation is important for maintaining homeostasis, while excessive inflammation can damage IECs, which is also a critical factor in the pathogenesis of UC. How to maintain the balance between inflammation and anti-inflammation of IECs may be question to investigate the pathogenesis of UC. In this study, we found that HSF2 can regulate mitophagy of IECs, inhibit inflammation of intestinal mucosal, and maintain intestinal homeostasis in UC. This study reveals a new regulatory mechanism for the intestinal mucosal homeostasis of HSF2 in UC.

Our study found that the number of damaged mitochondria in IECs of UC patients increased with disease severity, and the level of mtROS in intestinal mucosa increased continuously, which was consistent with previous studies (Liu et al., 2018), further proving the correlation between the level of damaged mitochondria and mtROS and the severity of UC. Therefore, timely removal of damaged mitochondria may be an important way to inhibit the inflammation of intestinal mucosa.

Mitophagy plays an important role in limiting the accumulation of mtROS and maintaining cell homeostasis by clearing damaged mitochondria (Larabi et al., 2020). In our study, we found that with increasing severity of UC, the level of mitophagy in IECs of UC patients rose accordingly. Similar results were found in mice with DSS-induced colitis and Caco-2 cells stimulated by LPS. This suggests that IECs may reduce mitochondrial injury under severe stress of UC by mitophagy, but it is not enough to reverse the disease process. These results are consistent with the physiological function of mitophagy and suggest that the level of mitophagy in IECs may be closely related to the activation of the NLRP3 inflammasome in UC, but the specific regulatory mechanism still needs to be further explored.

The signaling pathways of selective autophagy in damaged mitochondria have not been fully elucidated, including the PARL/PINK1/Parkin pathway (Dombi et al., 2018), the Nix protein mediated pathway (Zhang et al., 2019), and other pathways mediated by dynamic related protein 1 (Drp1) (Feng et al., 2020), among which the most recognized is the PARL/PINK1/Parkin pathway. Some studies have found that HSP72 regulated by HSF2 can regulate the level of mitophagy through Parkin protein (Kwong et al., 2006; Ostling et al., 2007; Ahn et al., 2008; Drew et al., 2014). Our study found that HSF2 may promote mitophagy in IECs through the PARL/PINK1/Parkin pathway and timely remove damaged mitochondria, thus reducing the accumulation of mtROS in cells and playing a protective role in UC.

Although we further elucidated the mechanism of HSF2 inhibiting inflammation of intestinal mucosa in UC, there are still some unanswered questions. As an important transcription factor of HSPs, HSF2 may play a role by regulating the expression level of HSPs. However, in our study, we did not continue to explore the relationship and regulatory mechanism between HSPs and the PARL/PINK1/Parkin pathway, mitophagy, and mtROS. In the future, we will further explore the specific relationship and potential regulatory mechanism of HSPs regulated by HSF2 with PARL/PINK1/Parkin pathway, mitophagy, mtROS and NLRP3 inflammasome in IECs, so as to lay a new theoretical foundation for further elucidating the mechanism of HSF2 in UC.

## Conclusion

In UC, HSF2 may promote mitophagy and decrease the level of mtROS through the PARL/PINK1/Parkin pathway. These results further elucidate the mechanism of specific high-level expression of HSF2 in UC alleviating the inflammation of intestinal mucosa.

## Data Availability

The original contributions presented in the study are included in the article/Supplementary Materials, further inquiries can be directed to the corresponding authors.

## References

[B1] Ahn J. Piri N. Caprioli J. Munemasa Y. Kim S. H. Kwong J. M. (2008). Expression of Heat Shock Transcription Factors and Heat Shock Protein 72 in Rat Retina after Intravitreal Injection of Low Dose N-Methyl-D-Aspartate. Neurosci. Lett. 433 (1), 11–16. 10.1016/j.neulet.2007.12.045 18242848

[B2] Ashrafi G. Schwarz T. L. (2013). The Pathways of Mitophagy for Quality Control and Clearance of Mitochondria. Cell Death Differ. 20 (1), 31–42. 10.1038/cdd.2012.81 22743996 PMC3524633

[B3] Chandraiah S. B. Ghosh S. Saha I. More S. S. Annappa G. S. Maiti A. K. (2021). Substance P Failed to Reverse Dextran Sulfate Sodium-Induced Murine Colitis Mediated by Mitochondrial Dysfunction: Implications in Ulcerative Colitis. 3 Biotech. 11 (4), 199. 10.1007/s13205-021-02755-2 PMC8006204 33927989

[B4] Dombi E. Mortiboys H. Poulton J. (2018). Modulating Mitophagy in Mitochondrial Disease. Curr. Med. Chem. 25 (40), 5597–5612. 10.2174/0929867324666170616101741 28618992

[B5] Drew B. G. Ribas V. Le J. A. Henstridge D. C. Phun J. Zhou Z. (2014). HSP72 Is a Mitochondrial Stress Sensor Critical for Parkin Action, Oxidative Metabolism, and Insulin Sensitivity in Skeletal Muscle. Diabetes 63 (5), 1488–1505. 10.2337/db13-0665 24379352 PMC3994950

[B6] Feng S. T. Wang Z. Z. Yuan Y. H. Wang X. L. Sun H. M. Chen N. H. (2020). Dynamin-related Protein 1: A Protein Critical for Mitochondrial Fission, Mitophagy, and Neuronal Death in Parkinson's Disease. Pharmacol. Res. 151, 104553. 10.1016/j.phrs.2019.104553 31760107

[B7] Geremia A. Biancheri P. Allan P. Corazza G. R. Di Sabatino A. (2014). Innate and Adaptive Immunity in Inflammatory Bowel Disease. Autoimmun. Rev. 13 (1), 3–10. 10.1016/j.autrev.2013.06.004 23774107

[B8] Guo W. Sun Y. Liu W. Wu X. Guo L. Cai P. (2014). Small Molecule-Driven Mitophagy-Mediated NLRP3 Inflammasome Inhibition Is Responsible for the Prevention of Colitis-Associated Cancer. Autophagy 10 (6), 972–985. 10.4161/auto.28374 24879148 PMC4091180

[B9] Kaplan G. G. Ng S. C. (2017). Understanding and Preventing the Global Increase of Inflammatory Bowel Disease. Gastroenterology 152 (2), 313–321.e2. 10.1053/j.gastro.2016.10.020 27793607

[B10] Kelley N. Jeltema D. Duan Y. He Y. (2019). The NLRP3 Inflammasome: An Overview of Mechanisms of Activation and Regulation. Int. J. Mol. Sci. 20 (13), 3328. 10.3390/ijms20133328 PMC6651423 31284572

[B11] Kudryavtseva A. V. Krasnov G. S. Dmitriev A. A. Alekseev B. Y. Kardymon O. L. Sadritdinova A. F. (2016). Mitochondrial Dysfunction and Oxidative Stress in Aging and Cancer. Oncotarget 7 (29), 44879–44905. 10.18632/oncotarget.9821 27270647 PMC5216692

[B12] Kwong J. M. Lalezary M. Nguyen J. K. Yang C. Khattar A. Piri N. (2006). Co-expression of Heat Shock Transcription Factors 1 and 2 in Rat Retinal Ganglion Cells. Neurosci. Lett. 405 (3), 191–195. 10.1016/j.neulet.2006.06.070 16889897

[B13] Larabi A. Barnich N. Nguyen H. T. T. (2020). New Insights into the Interplay between Autophagy, Gut Microbiota and Inflammatory Responses in IBD. Autophagy 16 (1), 38–51. 10.1080/15548627.2019.1635384 31286804 PMC6984609

[B14] Liu Z. Ren Z. Zhang J. Chuang C. C. Kandaswamy E. Zhou T. (2018). Role of ROS and Nutritional Antioxidants in Human Diseases. Front. Physiol. 9, 477. 10.3389/fphys.2018.00477 29867535 PMC5966868

[B15] Miao J. Niu J. Wang K. Xiao Y. Du Y. Zhou L. (2014). Heat Shock Factor 2 Levels Are Associated with the Severity of Ulcerative Colitis. PLoS One 9 (2), e88822. 10.1371/journal.pone.0088822 24533153 PMC3923051

[B16] Ng S. C. Shi H. Y. Hamidi N. Underwood F. E. Tang W. Benchimol E. I. (2017). Worldwide Incidence and Prevalence of Inflammatory Bowel Disease in the 21st Century: a Systematic Review of Population-Based Studies. Lancet 390 (10114), 2769–2778. 10.1016/S0140-6736(17)32448-0 29050646

[B17] Ordás I. Eckmann L. Talamini M. Baumgart D. C. Sandborn W. J. (2012). Ulcerative Colitis. Lancet 380 (9853), 1606–1619. 10.1016/S0140-6736(12)60150-0 22914296

[B18] Ostling P. Björk J. K. Roos-Mattjus P. Mezger V. Sistonen L. (2007). Heat Shock Factor 2 (HSF2) Contributes to Inducible Expression of Hsp Genes through Interplay with HSF1. J. Biol. Chem. 282 (10), 7077–7086. 10.1074/jbc.M607556200 17213196

[B19] Próchnicki T. Latz E. (2017). Inflammasomes on the Crossroads of Innate Immune Recognition and Metabolic Control. Cell Metab. 26 (1), 71–93. 10.1016/j.cmet.2017.06.018 28683296

[B20] Ranson N. Veldhuis M. Mitchell B. Fanning S. Cook A. L. Kunde D. (2018). NLRP3-Dependent and -Independent Processing of Interleukin (IL)-1β in Active Ulcerative Colitis. Int. J. Mol. Sci. 20 (1), 57. 10.3390/ijms20010057 PMC6337576 30583612

[B21] Sánchez de Medina F. Romero-Calvo I. Mascaraque C. Martínez-Augustin O. (2014). Intestinal Inflammation and Mucosal Barrier Function. Inflamm. Bowel Dis. 20 (12), 2394–2404. 10.1097/MIB.0000000000000204 25222662

[B22] Spoorthi B. C. More S. S. Gautham S. A. Ghosh S. Saha I. Maiti A. K. (2020). Role of Free Radical Scavenging Activity of Vasoactive Intestinal Peptide in the Attenuation of Mitochondrial Dysfunction to Ameliorate Dextran Sulphate Sodium-Induced Colitis in Mice: Implications in Ulcerative Colitis. J. Dig. Dis. 21 (12), 711–723. 10.1111/1751-2980.12932 33405317

[B23] Strowig T. Henao-Mejia J. Elinav E. Flavell R. (2012). Inflammasomes in Health and Disease. Nature 481 (7381), 278–286. 10.1038/nature10759 22258606

[B24] Swanson K. V. Deng M. Ting J. P. (2019). The NLRP3 Inflammasome: Molecular Activation and Regulation to Therapeutics. Nat. Rev. Immunol. 19 (8), 477–489. 10.1038/s41577-019-0165-0 31036962 PMC7807242

[B25] Wang W. Zhang F. Li X. Luo J. Sun Y. Wu J. (2020). Heat Shock Transcription Factor 2 Inhibits Intestinal Epithelial Cell Apoptosis through the Mitochondrial Pathway in Ulcerative Colitis. Biochem. Biophys. Res. Commun. 527 (1), 173–179. 10.1016/j.bbrc.2020.04.103 32446363

[B26] Wang Z. Li S. Cao Y. Tian X. Zeng R. Liao D. F. (2016). Oxidative Stress and Carbonyl Lesions in Ulcerative Colitis and Associated Colorectal Cancer. Oxid. Med. Cell Longev. 2016, 9875298. 10.1155/2016/9875298 26823956 PMC4707327

[B27] Yang Y. Wang H. Kouadir M. Song H. Shi F. (2019). Recent Advances in the Mechanisms of NLRP3 Inflammasome Activation and its Inhibitors. Cell Death Dis. 10 (2), 128. 10.1038/s41419-019-1413-8 30755589 PMC6372664

[B28] Yao D. Dong M. Dai C. Wu S. (2019). Inflammation and Inflammatory Cytokine Contribute to the Initiation and Development of Ulcerative Colitis and its Associated Cancer. Inflamm. Bowel Dis. 25 (10), 1595–1602. 10.1093/ibd/izz149 31287863

[B29] Zhang F. Zhao W. Zhou J. Wang W. Luo J. Feng Y. (2021). Heat Shock Transcription Factor 2 Reduces the Secretion of IL-1β by Inhibiting NLRP3 Inflammasome Activation in Ulcerative Colitis. Gene 768, 145299. 10.1016/j.gene.2020.145299 33181254

[B30] Zhang M. Shi R. Zhang Y. Shan H. Zhang Q. Yang X. (2019). Nix/BNIP3L-dependent Mitophagy Accounts for Airway Epithelial Cell Injury Induced by Cigarette Smoke. J. Cell Physiol. 234 (8), 14210–14220. 10.1002/jcp.28117 30618073 PMC13483001

[B31] Zhen Y. Zhang H. (2019). NLRP3 Inflammasome and Inflammatory Bowel Disease. Front. Immunol. 10, 276. 10.3389/fimmu.2019.00276 30873162 PMC6403142

[B32] Zuo T. Kamm M. A. Colombel J. F. Ng S. C. (2018). Urbanization and the Gut Microbiota in Health and Inflammatory Bowel Disease. Nat. Rev. Gastroenterol. Hepatol. 15 (7), 440–452. 10.1038/s41575-018-0003-z 29670252

