# Supplementary figures and images for: Heat Shock Transcription Factor 2 Promotes Mitophagy of Intestinal Epithelial Cells Through PARL/PINK1/Parkin Pathway in Ulcerative Colitis
Source: Front Pharmacol. 2022 Jul 4;13:893426. doi: 10.3389/fphar.2022.893426 (PMC9289131; doi:10.3389/fphar.2022.893426)

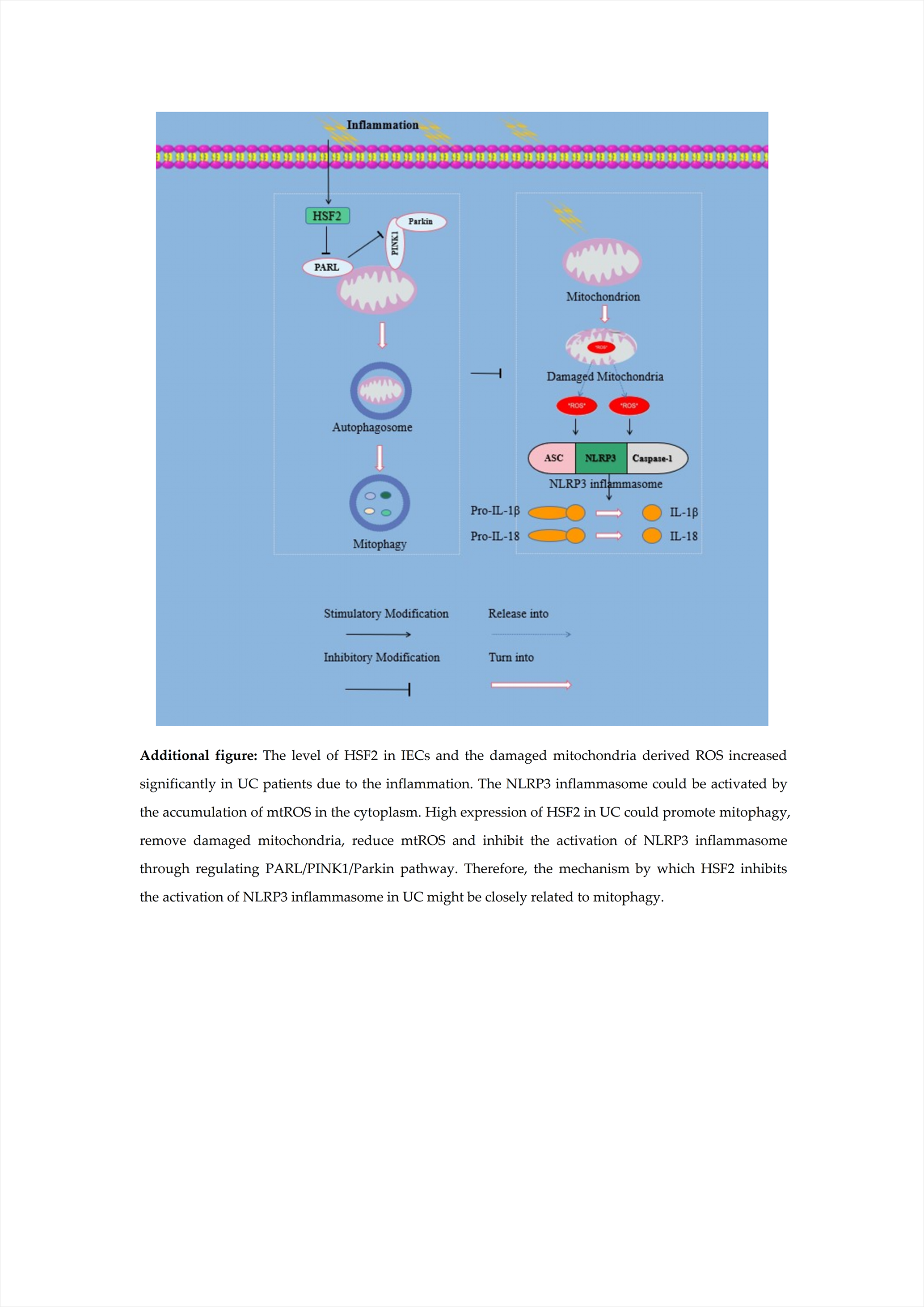

Supplement: Supplementary file 2 [file Image1.TIF]

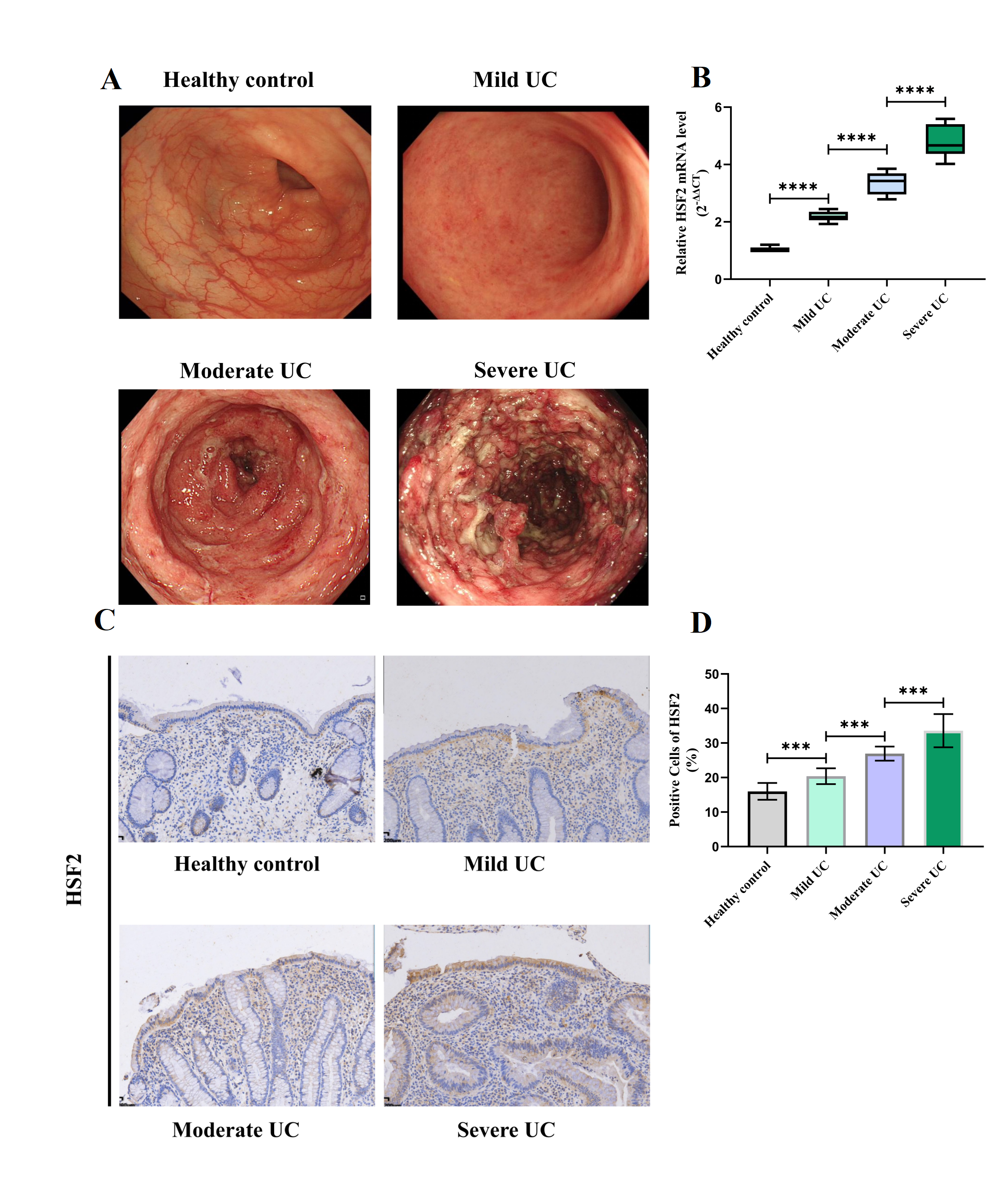

Supplement: Supplementary file 3 [file DataSheet2.ZIP › Supplementary Figures/Supplementary Figure 1.tif]

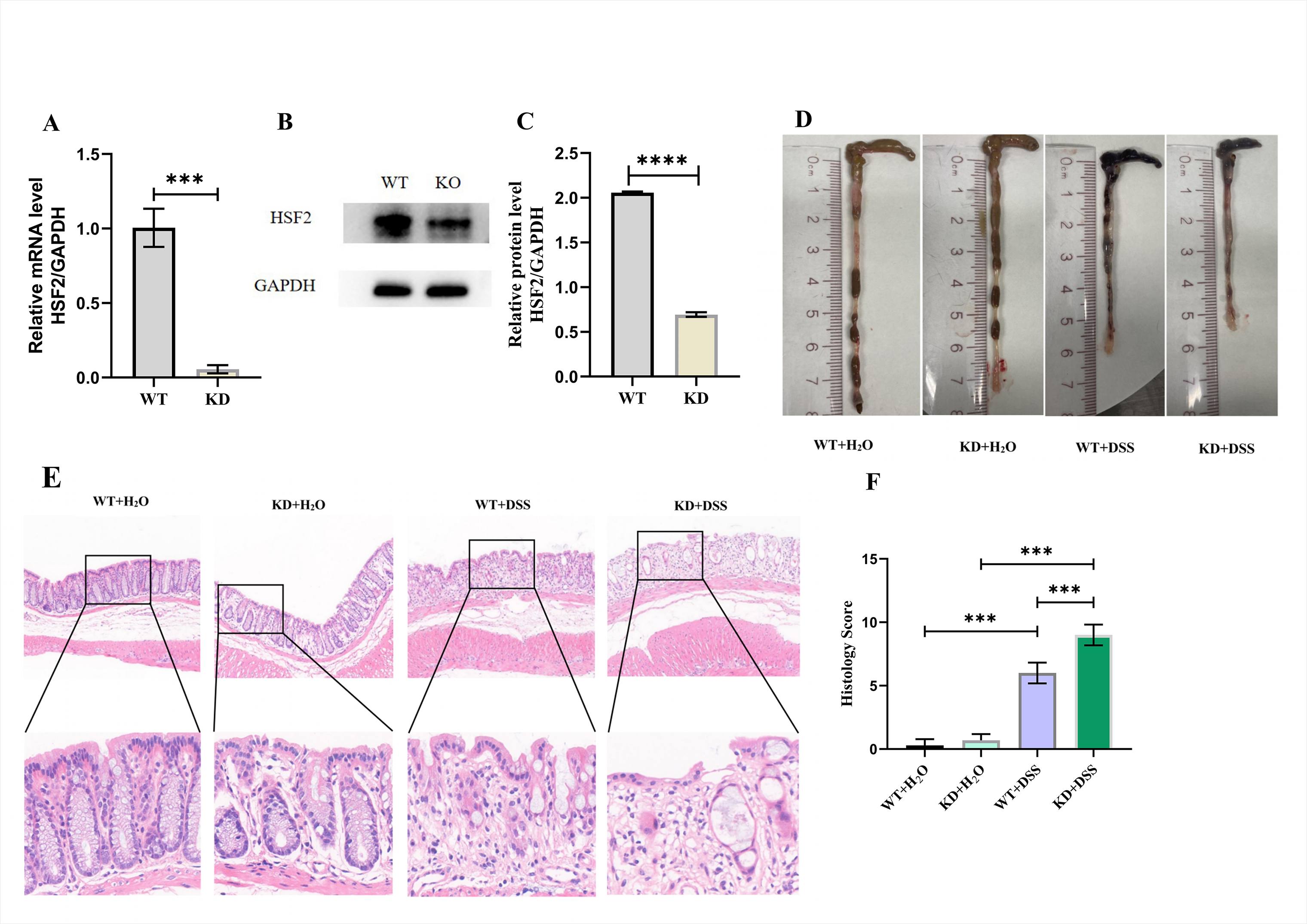

Supplement: Supplementary file 3 [file DataSheet2.ZIP › Supplementary Figures/Supplementary Figure 2.tif]

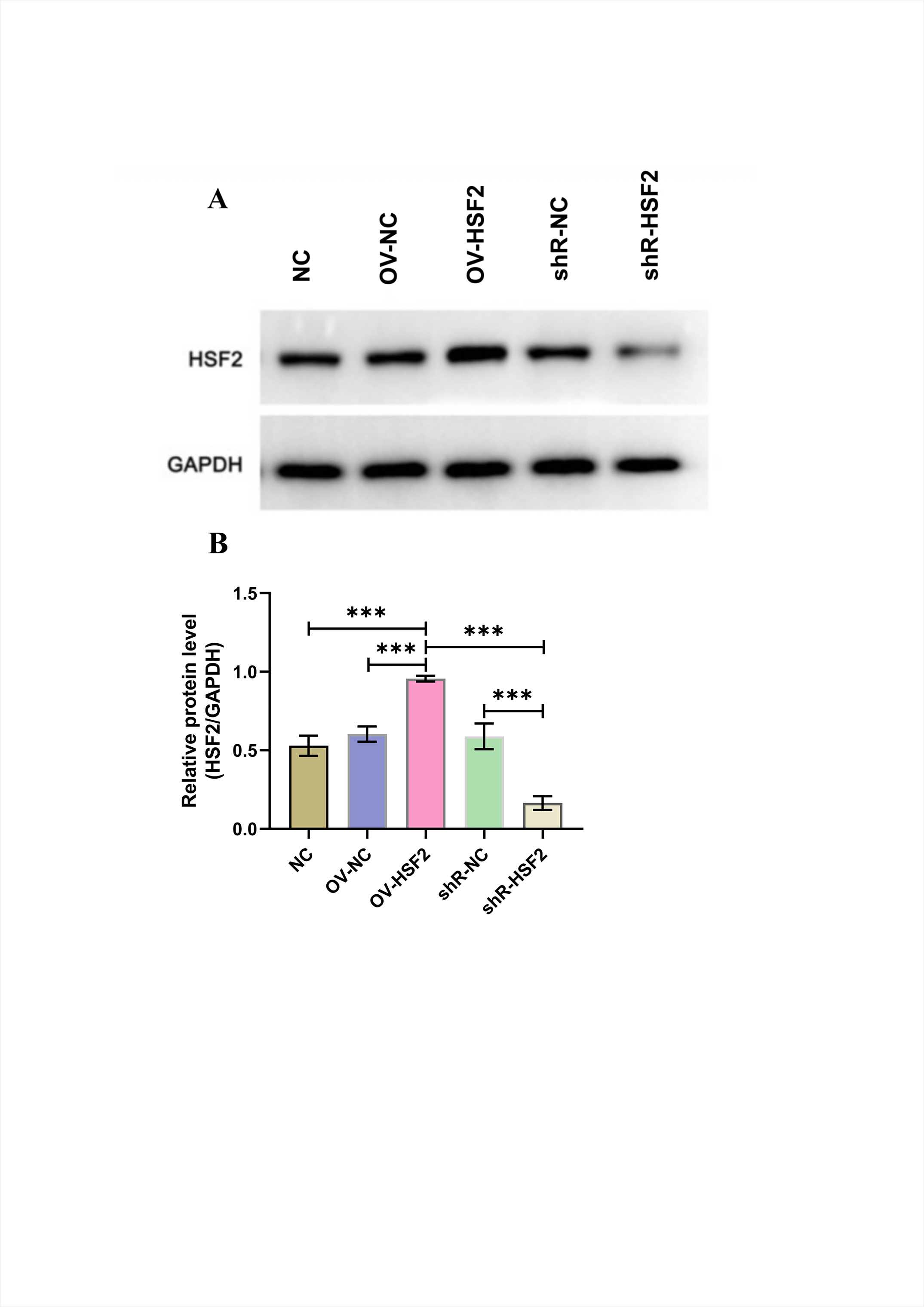

Supplement: Supplementary file 3 [file DataSheet2.ZIP › Supplementary Figures/Supplementary Figure 3.tif]

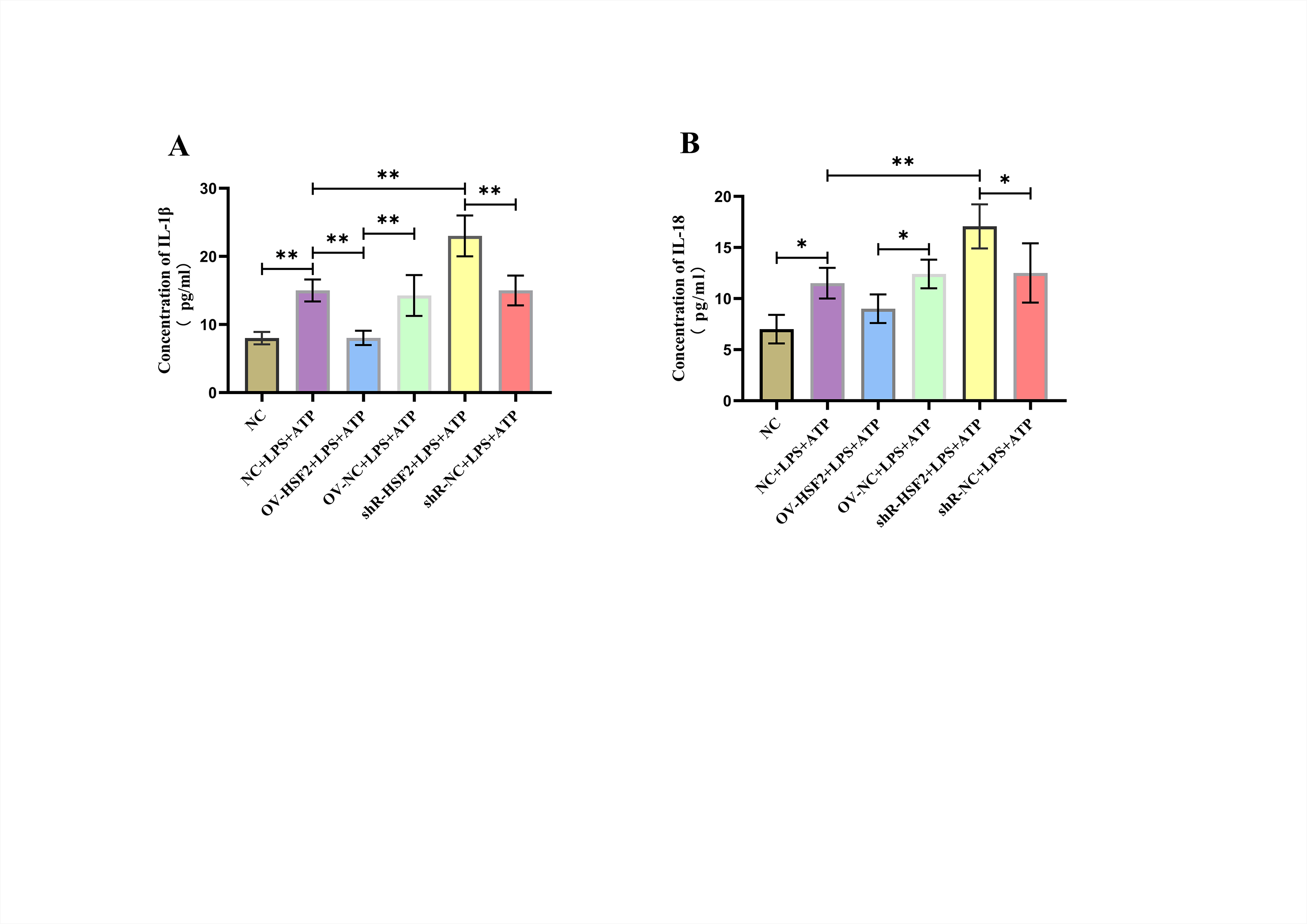

Supplement: Supplementary file 3 [file DataSheet2.ZIP › Supplementary Figures/Supplementary Figure 4.tif]
