# Supplementary material for: Heat Shock Transcription Factor 2 Promotes Mitophagy of Intestinal Epithelial Cells Through PARL/PINK1/Parkin Pathway in Ulcerative Colitis
Source: Front Pharmacol. 2022 Jul 4;13:893426. doi: 10.3389/fphar.2022.893426 (PMC9289131; doi:10.3389/fphar.2022.893426)
Supplement: Supplementary file 3 [file DataSheet2.ZIP › Supplementary Figures/Supplementary Figures.docx]

**Supplementary figure 1.** Compared with healthy controls, the expression level of HSF2 in the intestinal mucosa of UC patients was significantly increased, and was positively correlated with the severity of disease. (A) Colonoscopy of the rectum in healthy controls, mild, moderate and severe UC patients. (B) The expression level of HSF2 gene in intestinal mucosa of healthy controls and UC patients were measured by RT-PCR. (C, D) Immunohistochemistry was used to detect the level of HSF2 protein in intestinal mucosa of healthy controls and UC patients.

These data expressed as means ± standard deviation. Statistical significance at ****P*<0.001 and *****P*<0.0001.

**Supplementary figure 2.** Both wild-type and *hsf2^-/-^* mice showed colonic injury and inflammation after 7 days of DSS. And compared with the WT+DSS group, the KD+DSS group showed severer inflammation in colon tissue. There was no significant inflammation in the WT+H2O and KD+H2O groups (WT, wild-type mice; KD, *hsf2* knock down mice). (A) The expression level of *hsf2* gene in intestinal mucosa of mice were measured by RT-PCR. The expression of *hsf2* gene in KD group was significantly lower compared with the WT group. (B, C) The expression level of *hsf2* protein in intestinal mucosa of mice was detected by Western blotting. The expression of *hsf2* protein in KD group was significantly lower compared with the WT group. (D) Photos showed the colons of mice in each group. The colons of WT+DSS and KD+DSS groups were significantly shortened compared with the WT+H_2_O and KD+H_2_O groups. (E) HE staining showed the pathological results of colonic tissues in each group, WT+DSS and KD+DSS groups showed obvious inflammatory cell infiltration, epithelial cells loss and atrophy or disappearance of gland, and the KD+DSS group was more serious. (F) Compared with the WT+DSS group, the KD+DSS group showed higher histology score.

These data expressed as means ± standard deviation, and statistical results were obtained by one-way ANOVA. Statistical significance at ****P*<0.001 and *****P*<0.0001.

**Supplementary figure 3.** HSF2 gene level in Caco-2 cells was regulated by lentivirus. (A) The level of HSF2 protein in Caco-2 cells was detected by Western blotting. (NC, normal controls; OV-HSF2, HSF2 overexpression group; OV-NC, negative controls of overexpression; shR-HSF2, HSF2 knockdown group; shR-NC, negative controls of knockdown). (B) These data expressed as means ± standard deviation, and statistical results were obtained by one-way ANOVA. Statistical significance at ****P*<0.001.

**Supplementary figure 4.** The level of IL-1β and IL-18 in Caco-2 cells of each group was detected by ELISA and increased significantly after LPS and ATP were used to stimulate cells. In addition, after HSF2 overexpression, intracellular IL-1β and IL-18 decreased; on the contrary, after HSF2 downregulation, intracellular IL-1β and IL-18 increased, and difference was statistically significant. Statistical significance at **P*<0.05 and ***P*<0.01.
